# Real-world effectiveness and safety of trastuzumab-deruxtecan in Japanese patients with HER2-positive advanced gastric cancer (EN-DEAVOR study)

**DOI:** 10.1007/s10120-024-01555-w

**Published:** 2024-10-10

**Authors:** Hisato Kawakami, Koki Nakanishi, Akitaka Makiyama, Hirotaka Konishi, Satoshi Morita, Yukiya Narita, Naotoshi Sugimoto, Keiko Minashi, Motohiro Imano, Rin Inamoto, Yasuhiro Kodera, Hiroki Kume, Keita Yamaguchi, Wataru Hashimoto, Kei Muro, EN-DEAVOR Study Group

**Affiliations:** 1 Department of Medical Oncology, Kindai University Faculty of Medicine, Osakasayama, Japan; 2 Department of Gastroenterological Surgery, Nagoya University Graduate School of Medicine, Nagoya, Japan; 3 Cancer Center, Gifu University Hospital, Gifu, Japan; 4 Division of Digestive Surgery, Kyoto Prefectural University of Medicine, Kyoto, Japan; 5 Department of Biomedical Statistics and Bioinformatics, Kyoto University Graduate School of Medicine, Kyoto, Japan; 6 Department of Clinical Oncology, Aichi Cancer Center Hospital, Nagoya, Japan; 7 Department of Genetic Oncology, Osaka International Cancer Institute, Osaka, Japan; 8 Division of Gastroenterology, Chiba Cancer Center, Chiba, Japan; 9 Department of Surgery, Kindai University Faculty of Medicine, Osakasayama, Japan; 10 Department of Gastroenterology, Saitama Cancer Center, Saitama, Japan; 11 Oncology Medical Science Department I, Daiichi Sankyo Co. Ltd., Tokyo, Japan; 12 Data Intelligence Department, Daiichi Sankyo Co. Ltd., Tokyo, Japan

**Keywords:** Effectiveness, Gastric cancer, HER2-positive, Japan, Post-marketing surveillance, Real world, Safety, Third- or later-line, Trastuzumab-deruxtecan (T-DXd)

## Abstract

**Background:**

Trastuzumab-deruxtecan (T-DXd) was approved for the treatment of HER2-positive patients with advanced gastric cancer in Japan based on the results of the DESTINY-Gastric01 trial. This study aimed to collect real-world data and evaluate the effectiveness and safety of T-DXd.

**Methods:**

Patients aged ≥ 20 years at the start of T-DXd administration with a histopathologically confirmed diagnosis of HER2-positive unresectable advanced or recurrent gastric or gastroesophageal junction (GEJ) adenocarcinoma that had worsened after chemotherapy were enrolled in this retrospective cohort study. Key outcomes included T-DXd treatment status, overall survival (OS), real-world progression-free survival (rwPFS), time to treatment failure (TTF), objective response rate and frequency of grade ≥ 3 adverse events (AEs).

**Results:**

Of the 312 patients included in the analysis, 75.3% were male, the median (range) age was 70.0 (27.0–89.0) years, 12.2% had an ECOG PS ≥ 2, 43.3% had ascites and the initial T-DXd dose was > 5.4– ≤ 6.4 mg/kg in 78.2% of patients. The median (95% confidence interval) OS, rwPFS and TTF (months) was 8.9 (8.0–11.0), 4.6 (4.0–5.1) and 3.9 (3.4–4.2), respectively. The response rate was 42.9% in patients with a target lesion. In total, 48.4% of patients experienced a grade ≥ 3 AE, 2.6% experienced grade 5 AEs and 60.9% experienced AEs leading to T-DXd dose adjustments (reduction: 36.9%, interruption: 34.0% or discontinuation: 23.7%). No new safety signals were detected.

**Conclusions:**

T-DXd was effective and had a manageable safety profile as a third- or later-line treatment for patients with HER2-positive gastric or GEJ cancer in Japanese clinical practice. Clinical trial registration: UMIN000049032

**Supplementary Information:**

The online version contains supplementary material available at 10.1007/s10120-024-01555-w.

## Introduction

Globally, gastric cancer was the fifth most diagnosed cancer and the fourth leading cause of cancer-related deaths in 2020 [1]. In Japan, gastric cancer accounted for 12.6% of all cancers and was the third leading cause of cancer-related deaths in 2022 [2]. The 5-year relative survival rate during 2009–2011 in Japan was 66.6% (67.5% for men and 64.6% for women) [3].

Human epidermal growth factor receptor 2 (HER2) is overexpressed/amplified in approximately 20% of gastric or gastroesophageal junction (GEJ) cancers and contributes to a poor prognosis [4, 5]. Between September 2017 and March 2020, the most commonly prescribed monotherapy for third- or later-line treatment in Japanese patients with HER2-positive, unresectable, recurrent or metastatic gastric cancer was nivolumab followed by irinotecan and trifluridine/tipiracil, with a median (95% confidence interval [CI]) overall survival (OS) of 6.2 (4.5–8.0) months [6]. Trastuzumab-deruxtecan (T-DXd), an antibody–drug conjugate, has demonstrated efficacy and safety in patients with HER2-positive and HER2-low gastric or GEJ cancer who had progressed despite receiving previous therapies, including trastuzumab [7–10]. In the phase 2, open-label, DESTINY-Gastric01 trial, the objective response rate (ORR) of the HER2-positive cohort was 51% and 14% with T-DXd and chemotherapy, respectively (P < 0.001) [9].

T-DXd was approved for HER2-positive patients with advanced gastric cancer who had received prior treatment in September 2020 in Japan [11]. T-DXd is recommended by the 2023 National Comprehensive Cancer Network (NCCN) gastric cancer guidelines as a second-line or subsequent treatment option for patients with HER2 overexpression–positive adenocarcinoma following failure of prior treatment with a trastuzumab-based regimen [12]. The 2021 Japanese Gastric Cancer Association (JGCA) Treatment Guidelines recommend that T-DXd should be prioritised as a third-line therapy for patients with HER2-positive gastric cancer, as it is the only treatment for which survival prolongation was confirmed in comparison with chemotherapy regimens [13]. So far, real-world studies of nivolumab in Japanese patients with advanced gastric cancer have reported a median OS of 5.8–7.5 months (n = 487 and n = 70, respectively) [14, 15]. In addition, there are 2 Japanese real-world studies of T-DXd in HER2 positive advanced gastric cancer published till date; however, the sample sizes were small (n = 18 and n = 20) [16, 17]. This study aimed to evaluate the real-world effectiveness and safety of T-DXd in a large number of patients with HER2-positive, unresectable, advanced or recurrent gastric cancer (gastric adenocarcinoma or GEJ adenocarcinoma).

## Methods

### Study design and patients

This was a multi-institutional retrospective cohort study (UMIN000049032) conducted across 63 sites that enrolled patients aged ≥ 20 years when T-DXd was first administered; patients with a histopathologically confirmed diagnosis of HER2-positive (immunohistochemistry [IHC] 3 + or IHC 2 + with in situ hybridisation [ISH] +) unresectable advanced or recurrent gastric or GEJ adenocarcinoma that had worsened after chemotherapy and had started treatment with T-DXd between 25 September 2020 and 30 September 2021 were eligible. In principle, HER2 testing had to be conducted before introduction of the first-line treatment in which case re-evaluation of the HER2 status prior to T-DXd administration was not mandatory. At the participating sites, it was mandatory to register all patients who met the inclusion criteria; the inclusion criteria for DESTINY-Gastric01 are shown in Supplementary Table 1. Patients who refused to participate, had other primary malignancies that the investigator determined would affect the evaluation of T-DXd or had a history of T-DXd administration in other interventional studies (including clinical trials and expanded access programmes) or at other sites were not included in this study. Eligible patients were followed up until 30 September 2022 to enable a minimum of 12 months of observation.

### Outcomes

Data on demographics and clinical characteristics before T-DXd administration and on T-DXd treatment status during each cycle were collected. Effectiveness outcomes included OS, real-world progression-free survival (rwPFS), time to treatment failure (TTF; defined as the interval from start date [date of “first T-DXd dose”] to premature discontinuation [date of event “any death” and “treatment discontinuation”, date of censoring ‘date of last dose’]), ORR and disease control rate (DCR) in patients with a target lesion, best overall response (BOR) and best percent change from baseline in the sum of tumour diameters (patients with target lesions). Data on the frequencies of grade ≥ 3 adverse events (AEs) and AEs leading to T-DXd dose reduction, discontinuation or interruption were also collected. AEs were graded using the National Cancer Institute Common Terminology Criteria for Adverse Events, version 5.0.

### Statistical analysis

The sample size was set to 300 patients considering the data collection feasibility at the participating study sites. The analysis population comprised all patients registered for the study, except those with significant protocol deviations and those deemed ineligible at the case-review meeting. Descriptive statistics were used for categorical and continuous variables. A subgroup analysis was conducted for OS, rwPFS and ORR in patients with target lesions. In another subgroup analysis, OS and rwPFS were evaluated over 2 years among patients who continued to receive T-DXd after the 3rd cycle and (1) experienced AEs leading to dose reduction, interruption or discontinuation, or (2) experienced grade ≥ 3 AEs. Additional analysis was conducted for patients whose dosing was compliant with the package insert for T-DXd (for example, 6.4 mg/kg given as intravenous infusion once every 3 weeks until disease progression or unacceptable toxicity). No imputation was conducted for the missing data. Statistical analyses were performed using SAS software version 9.4.

## Results

### Patient disposition

A total of 318 patients were enrolled in this study, of whom 312 were analysed; 6 patients were excluded owing to incorrect enrolment (double registration). T-DXd was discontinued in 299 (95.8%) patients due to progressive disease (PD, 72.1%), AEs (23.7%) and other reasons (3.2%).

### Demographics and baseline clinical characteristics

Among the 312 patients, 75.3% were male, the median (range) age was 70.0 (27.0–89.0) years and 12.2% had an Eastern Cooperative Oncology Group performance status (ECOG PS) of ≥ 2. The HER2 status (IHC and ISH) before T-DXd treatment was as follows: IHC 3 + (69.6%), IHC ‍2 + and ISH + (27.2%) and others (3.2%). The site of the primary lesion was the stomach (84.6%) and GEJ (15.4%). A total of 61.5% of the patients had metastasis in ≥ 2 organs, and the most common metastatic sites were the lymph nodes (65.7%). Ascites was present in 43.3% of patients, and 25.0% had received ≥ 4 lines of previous therapies; trastuzumab and nivolumab were used in 92.3% and 40.7% of the patients, respectively (Table 1).

**Table 1:** Demographics and baseline clinical characteristics

Patient characteristics	N = 312
Sex	
Male	235 (75.3)
Female	77 (24.7)
Age (years), median (range)	70.0 (27.0–89.0)
ECOG PS	
0–1	272 (87.2)
≥ 2	38 (12.2)
Unknown	2 (0.6)
HER2 status (IHC and ISH): Before T-DXd treatment^a^	
IHC 3 +	217 (69.6)
IHC 2 + and ISH +	85 (27.2)
Others^b^	10 (3.2)
Site of primary lesions	
Stomach	264 (84.6)
Gastroesophageal junction	48 (15.4)
Any surgeries for primary lesions	
No	205 (65.7)
Histological type of primary lesions	
Diffuse	79 (25.3)
Intestinal	170 (54.5)
Others	21 (6.7)
Unknown	42 (13.5)
Number of metastatic organs	
0	1 (0.3)
1	119 (38.1)
≥ 2	192 (61.5)
Metastasis site	
Lymph nodes	205 (65.7)
Liver	159 (51.0)
Peritoneum	115 (36.9)
Lung	69 (22.1)
Bones	29 (9.3)
Brain	9 (2.9)
Others	25 (8.0)
Ascites	
Yes	135 (43.3)
Presence of measurable lesions	
Yes	226 (72.4)
Number of lines of previous therapy	
1	5 (1.6)
2	156 (50.0)
3	73 (23.4)
≥ 4	78 (25.0)
Previous therapies^c^	
Taxane	290 (92.9)
Paclitaxel	149 (47.8)
Nab-paclitaxel	140 (44.9)
Docetaxel	15 (4.8)
HER2 inhibitors	
Trastuzumab	288 (92.3)
Immune checkpoint inhibitors	131 (42.0)
Nivolumab	127 (40.7)
Pembrolizumab	5 (1.6)
Angiogenesis inhibitors	
Ramucirumab	256 (82.1)
Platinum	280 (89.7)
Cisplatin	78 (25.0)
Oxaliplatin	225 (72.1)
Irinotecan hydrochloride hydrate	49 (15.7)
Pyrimidine fluoride	296 (94.9)
Capecitabine	124 (39.7)
Tegafur-uracil	2 (0.6)
Tegafur-gimeracil-oteracil potassium	191 (61.2)
Fluorouracil	21 (6.7)
Calcium levofolinate	15 (4.8)
Others	42 (13.5)
Trifluridine/tipiracil hydrochloride (TAS-102)	35 (11.2)
Investigational drugs	6 (1.9)
Panitumumab (genetical recombination)	1 (0.3)
Trastuzumab treatment history^c^	
Duration of trastuzumab treatment before T-DXd treatment (months), median (range)	6.4 (0.0–81.5)
Trastuzumab-free interval (months), median (range)	6.8 (0.1–70.6)
Nivolumab treatment history^c^	
Duration of nivolumab treatment before T-DXd treatment (months), median (range)	2.4 (0.0–26.9)
Time from final dose of nivolumab to the first dose of T-DXd treatment (months), median (range)	2.1 (0.1–28.6)

Data are presented as n (%) unless otherwise specified

*ECOG PS* Eastern Cooperative Oncology Group performance status, *HER2* human epidermal growth factor receptor type 2, *IHC* immunohistochemistry, *ISH* in situ hybridisation, *NGS* next-generation sequencing, *T-DXd* trastuzumab-deruxtecan

^a^According to the HER2 status survey items at the time of initial diagnosis of gastric cancer, 299/312 patients were enrolled as HER2 positive based on IHC and ISH investigation data, and 13/312 patients were HER2 negative or information on HER2 testing was not available. Of these 13 patients, 3 were confirmed as HER2 positive prior to T-DXd dosing, making the total number of HER2-positive patients 302 (IHC 3 + : 217 and ISH 2 + and ISH + : 85)

^b^Four patients were confirmed to be eligible for treatment with T-DXd using NGS. The remaining 6 patients were confirmed to be HER2 positive and eligible for T-DXd treatment by the diagnosing physician; however, IHC and ISH information for these patients were unknown

^c^For patients with a history of > 1 previous treatment, the last data were tabulated

### Treatment status of T-DXd

**Fig. 1:**
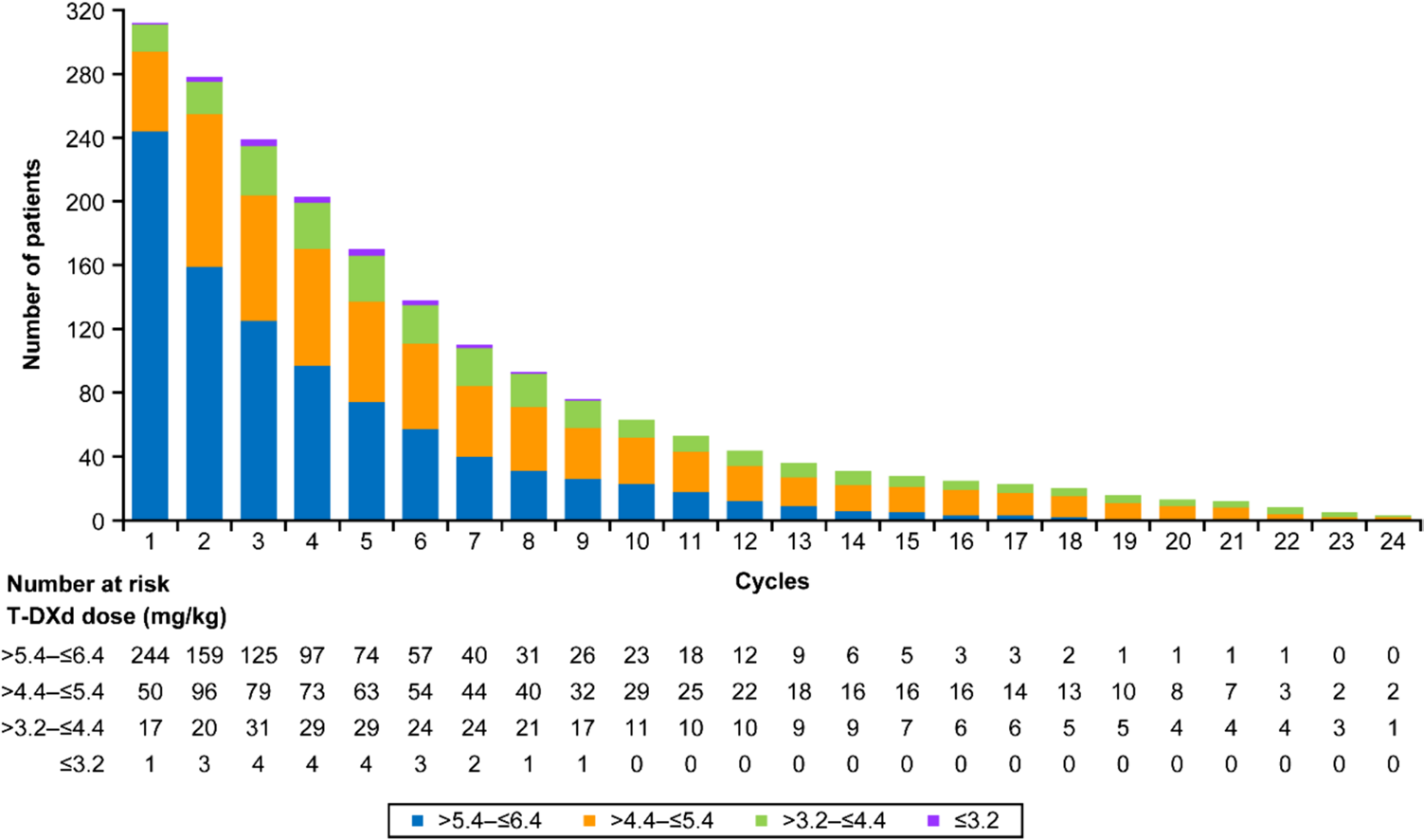
Treatment status of T-DXd. T-DXd, trastuzumab-deruxtecan

Approximately one-third of patients received < 4 cycles of T-DXd (34.9%), followed by ≥ 4– < 7 cycles (29.8%), ≥ 7– < 10 cycles (15.1%), ≥ 10– < 13 cycles (8.7%), ≥ 16 cycles (8.0%) and ≥ 13–‍ < 16 cycles (3.5%). The initial dose of T-DXd (mg/kg) was the normal dose of > 5.4– ≤ 6.4 in 244 (78.2%) and 1-step dose reduction dosage of > 4.4– ≤ 5.4 in 50 (16.0%) patients (Fig. 1).

### Effectiveness outcomes

**Fig. 2:**
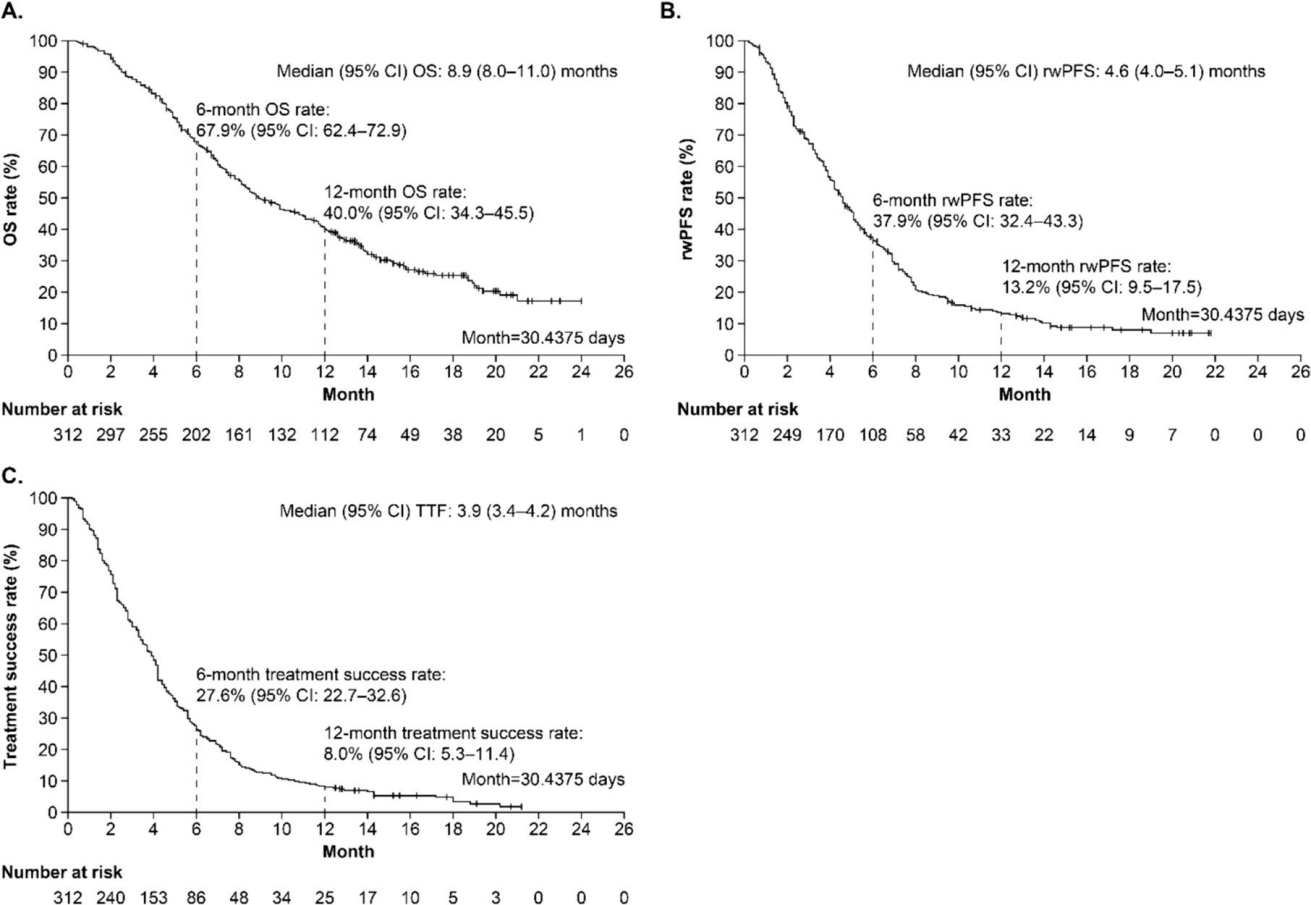
Survival outcomes of T-DXd: **A** OS rate, **B** rwPFS rate and (**C**). TTF rate. The median (95%) CI time was determined using the Brookmeyer and Crowley method. *CI* confidence interval, *OS* overall survival, *rwPFS* real-world progression-free survival, *TTF* time to treatment failure, *T-DXd* trastuzumab-deruxtecan

The median follow-up period was 8.3 months. The median (95% CI) OS, rwPFS and TTF (months) was 8.9 (8.0–11.0), 4.6 (4.0–5.1) and 3.9 (3.4–4.2), respectively (Fig. 2).

**Fig. 3:**
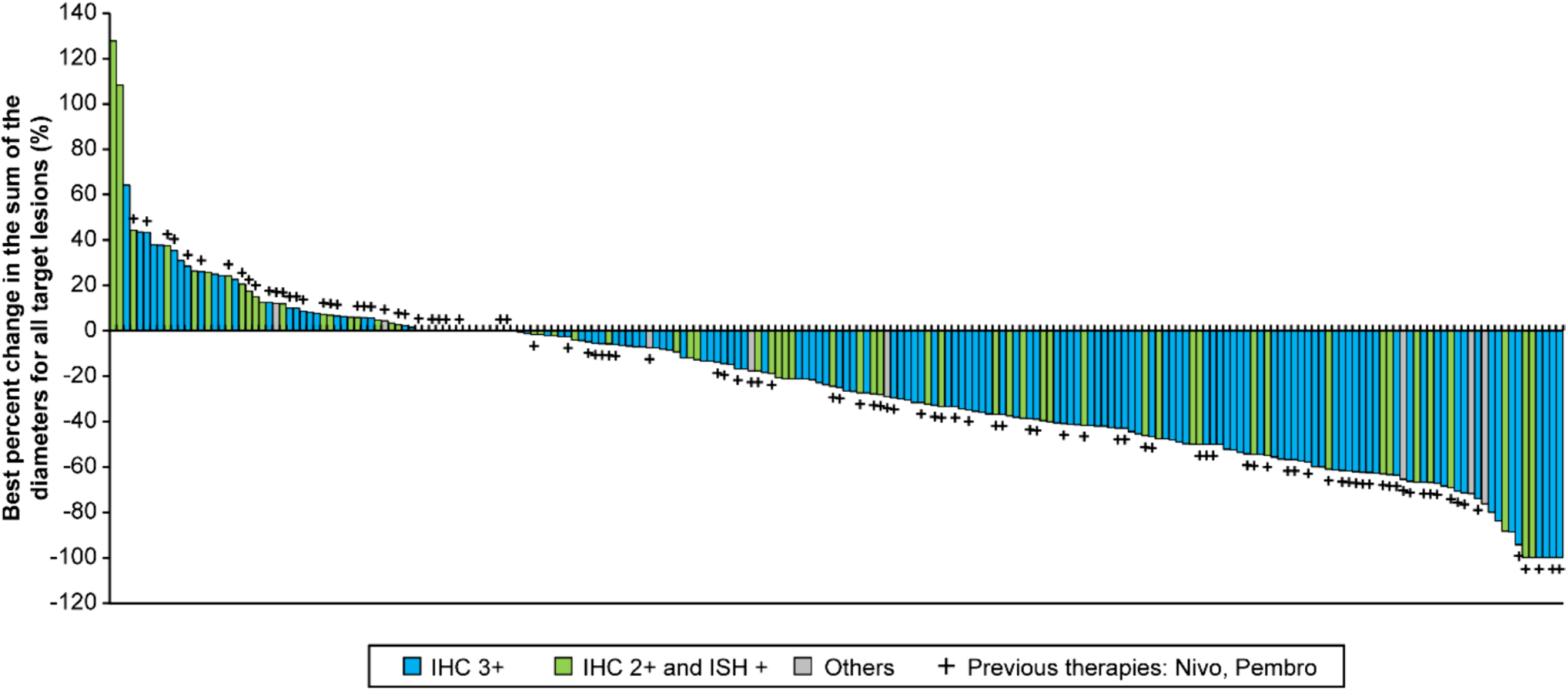
Best percentage change from baseline in sum of diameters of target lesions. *IHC* immunohistochemistry, *ISH* in situ hybridisation, Nivo, nivolumab; Pembro, pembrolizumab

The response rate was 42.9% in patients with a target lesion (n = 226; Supplementary Table 2), and 32.1% of patients had an objective response overall. The DCR was 81.4% (95% CI: 75.7–86.3) in patients with a target lesion. The BOR in patients with a target lesion was complete response (CR) in 2.2%, partial response (PR) in 40.7%, stable disease (SD) in 38.5%, PD in 13.7% and not evaluable in 4.9% of patients (Supplementary Table 2). The median (range) sum of diameters of the baseline tumour was 45.5 (10.0–229.7) mm, and the best percentage change from baseline in the sum of diameters for all target lesions was − 24.7% (− 100.0 to 127.7; Fig. 3).

### Safety outcomes

A total of 151 (48.4%) patients experienced a grade ≥ 3 AE (grade 3: 118 [37.8%]; grade 4: 25 [8.0%]; grade 5: 8 [2.6%]). Of the 8 patients who experienced grade 5 AEs, 5 had interstitial pneumonia and 1 each had febrile neutropenia, pneumonia and pneumonia caused by cytomegalovirus (Table 2). Overall, 60.9% of patients experienced AEs that led to T-DXd dose adjustments; dose reduction (36.9%), interruption (34.0%) or discontinuation (23.7%; Supplementary Table 3).

**Table 2:** Frequency of grade ≥ 3 AEs

	Grade ≥ 3	Grade 3	Grade 4	Grade 5
Any AE	151 (48.4)	118 (37.8)	25 (8.0)	8 (2.6)
Haematotoxicity	89 (28.5)	70 (22.4)	19 (6.1)	0 (0.0)
Non-haematotoxicity	88 (28.2)	73 (23.4)	7 (2.2)	8 (2.6)
Nausea	13 (4.2)	13 (4.2)	0 (0.0)	0 (0.0)
Vomiting	6 (1.9)	6 (1.9)	0 (0.0)	0 (0.0)
Anorexia	29 (9.3)	29 (9.3)	0 (0.0)	0 (0.0)
Malaise	14 (4.5)	14 (4.5)	0 (0.0)	0 (0.0)
Anaemia	29 (9.3)	28 (9.0)	1 (0.3)	0 (0.0)
Diarrhoea	5 (1.6)	5 (1.6)	0 (0.0)	0 (0.0)
Fatigue	3 (1.0)	3 (1.0)	0 (0.0)	0 (0.0)
Neutrophil count decreased	61 (19.6)	46 (14.7)	15 (4.8)	0 (0.0)
Platelet count decreased	13 (4.2)	9 (2.9)	4 (1.3)	0 (0.0)
White blood cell decreased	10 (3.2)	10 (3.2)	0 (0.0)	0 (0.0)
Lymphocyte count decreased	1 (0.3)	1 (0.3)	0 (0.0)	0 (0.0)
Febrile neutropenia	4 (1.3)	3 (1.0)	0 (0.0)	1 (0.3)
Interstitial pneumonia	14 (4.5)	8 (2.6)	1 (0.3)	5 (1.6)
Pneumonitis	5 (1.6)	3 (1.0)	2 (0.6)	0 (0.0)
Others	27 (8.7)	20 (6.4)	5 (1.6)	2 (0.6)^a^

Data are presented as n (%)

*AE* adverse event

^a^Pneumonia (n = 1) and pneumonia caused by cytomegalovirus (n = 1)

### Subgroup analysis

**Fig. 4:**
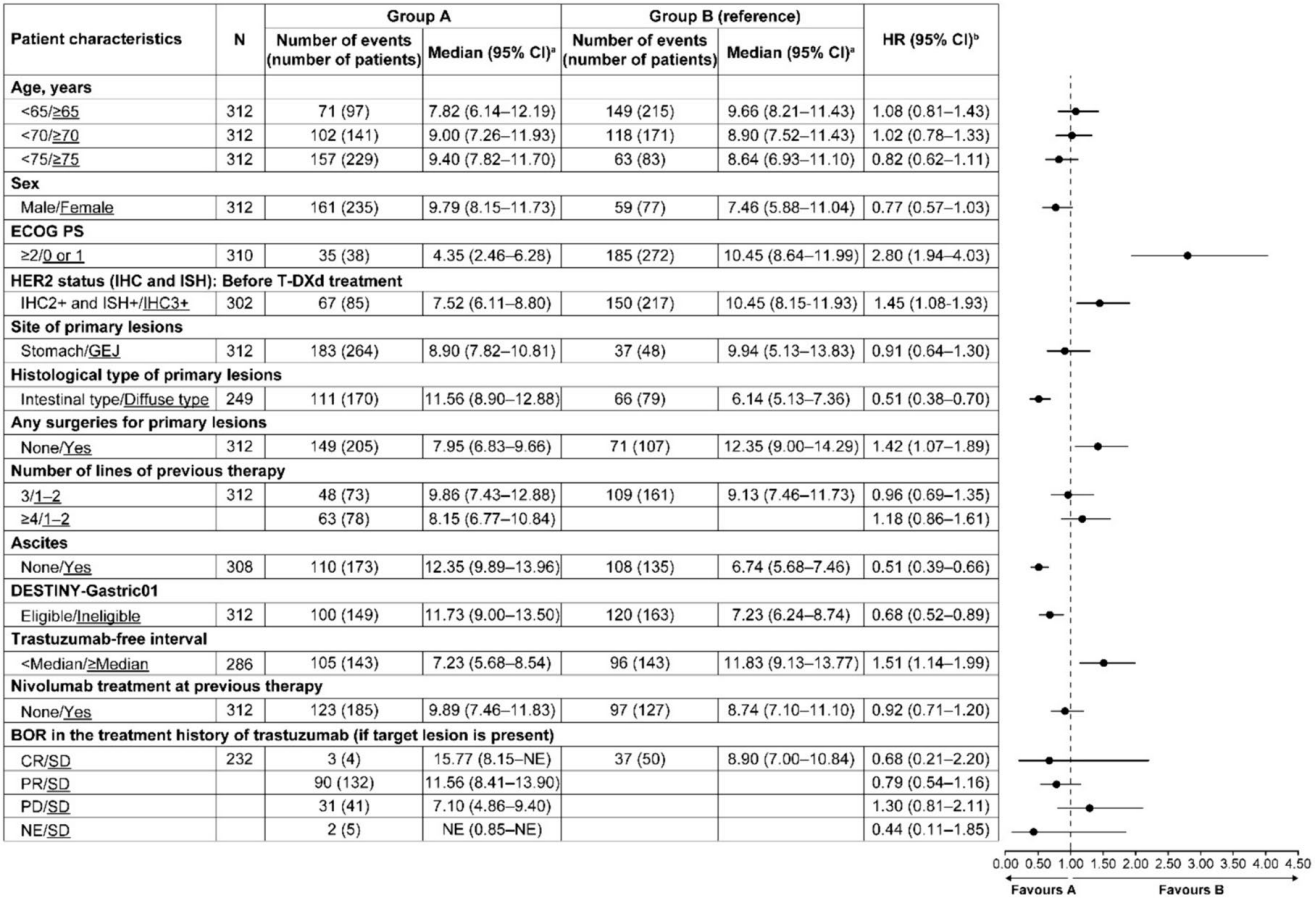
Subgroup analysis of OS. The underlined values are for Group B (reference). ^a^Brookmeyer and Crowley method. ^b^Using the univariate Cox’s proportional hazard model. *BOR* best overall response, *CI* confidence interval, *CR* complete response, *ECOG PS* Eastern Cooperative Oncology Group performance status, *GEJ* gastroesophageal junction, *HER2* human epidermal growth factor receptor type 2, *HR* hazard ratio, *IHC* immunohistochemistry, *ISH* in situ hybridisation, *NE* not estimable, *OS* overall survival, *PD* progressive disease, *PR* partial response, *SD* stable disease, *T-DXd* trastuzumab deruxtecan

**Fig. 5:**
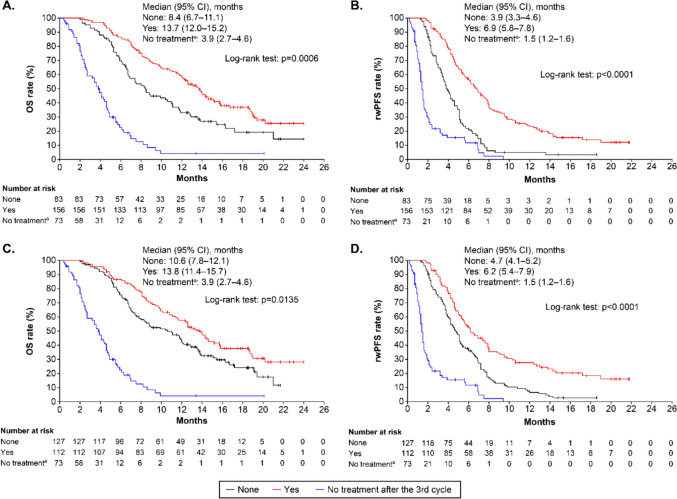
Survival rates of patients requiring dose adjustment and those who experienced grade ≥ 3 AEs after having completed 3 cycles of T-DXd. **A** OS in patients who experienced AEs that led to dose reduction, postponement or discontinuation of T-DXd under T-DXd treatment, **B** rwPFS in patients who experienced AEs that led to dose reduction, postponement or discontinuation of T-DXd under T-DXd treatment, **C** OS in patients who experienced grade ≥ 3 AEs under T-DXd treatment, **D** rwPFS in patients who experienced grade ≥ 3 AEs under T-DXd treatment. 1 month = 30.4375 days. ^a^T-DXd was discontinued within 2 cycles. The median (95% CI) time was determined using the Brookmeyer and Crowley method. *AE* adverse event, *CI* confidence interval, *OS* overall survival, *rwPFS* real-world progression-free survival, *T-DXd* trastuzumab-deruxtecan

Patients with ECOG PS ≥ 2, HER2 IHC 2 + and ISH + , those who had not undergone any surgery for primary lesions and trastuzumab-free interval < median had a significantly shorter OS compared with those who had ECOG PS 0 or 1, HER2 IHC 3 + , undergone any surgery for the primary lesions and trastuzumab-free interval ≥ median, respectively. Patients with intestinal type of primary lesions, without ascites, eligible for DESTINY-Gastric01 had a significantly longer OS than those with diffuse lesions, patients with ascites and patients ineligible for DESTINY-Gastric01, respectively (Fig. 4). Similar results were noted for rwPFS; furthermore, patients who had BOR of PR in the treatment history of trastuzumab (if target lesion was present) had a significantly longer rwPFS than those with BOR of SD (Supplementary Fig. 1). Patients with HER2 IHC3 + status had significantly better ORR for target lesions than those with HER2 IHC2 + and ISH + status (Supplementary Fig. 2). At the 2-year follow-up, the median OS and rwPFS rates were significantly longer among those who experienced AEs that led to dose reduction, interruption or discontinuation after having completed 3 cycles of T-DXd compared with those who did not experience these AEs and those who did not receive T-DXd after the 3rd cycle (Fig. 5a and b). Similar results were noted for grade ≥ 3 AEs after having completed 3 cycles of T-DXd (Fig. 5c and d).

Another subgroup analysis revealed that the median OS increased with an increase in the best percent change from baseline in the sum of diameters for all target lesions (≤ –80: not reached and ≥ 20: 5.4 months) (Supplementary Fig. 3).

### Package insert−compliant population

The results in the package insert (PI)−compliant population were generally similar to those of the overall population (Supplementary material).

## Discussion

This study highlights the effectiveness and safety of T-DXd in a real-world setting in Japanese patients with HER2-positive unresectable advanced or recurrent gastric or GEJ cancer. The median OS and median rwPFS in this study were 8.9 months and 4.6 months, respectively, suggesting that T-DXd is an effective treatment for HER2-positive gastric cancer at third- or later-line in clinical practice. Apart from the AEs reported in DESTINY-Gastric01, no new AEs were reported in this study [9]. Overall, the findings of this real-world study confirm the effectiveness of T-DXd in patients with HER2-positive advanced gastric cancer with a manageable safety profile.

The median age of patients included in this study was higher than that of those included in DESTINY-Gastric01 (70.0 years vs. 65.0 years) [9]. Patients with an ECOG PS of ≥ 2 were excluded from DESTINY-Gastric01 [9]; however, this study included 12.2% of patients with an ECOG PS ≥ 2. Moreover, a substantial proportion of patients had ascites or peritoneal metastasis, which have been negatively associated with overall therapy duration [18]. The demographics and clinical characteristics of patients in the current study were generally similar to other Japanese real-world studies [6, 14, 17]. Thus, the current study population represents a heavily treated group of patients with a metastatic pattern that tends to respond poorly to conventional treatments and is a good reflection of real-world clinical practice of gastric cancer in Japan.

The median OS in this study was 8.9 months, whereas the median OS was 12.5 months in DESTINY-Gastric01 [9]. The median rwPFS in patients treated with T-DXd in this study was also shorter than the progression-free survival (PFS) in DESTINY-Gastric01 (4.6 months vs. 5.6 months) [9]. The ORR was also lower in this study compared with DESTINY-Gastric01 (42.9% vs. 51%) [9]. Thus, the effectiveness outcomes were numerically lower in this study than in DESTINY-Gastric01; however, it is important to understand that patients in this study had more advanced disease or were ineligible for participation in DESTINY-Gastric01 (12.2% of patients with an ECOG PS ≥ 2) and had a higher median age (70.0 years vs. 65.0 years) [9]. Therefore, comparisons of the survival outcomes of the current study with DESTINY-Gastric01 should be done cautiously [9]. On the other hand, the ORR with T-DXd in this study was substantially higher than that obtained with nivolumab, irinotecan and trifluridine/tipiracil in the third- or later-line treatment of patients with advanced gastric cancer in Japan (42.9% vs. 0–9.2%) [6]. These data suggest the robust anticancer effect of T-DXd in patients with advanced gastric cancer in real-world clinical practice. In DESTINY-Gastric01, > 80% of patients who received T-DXd had a reduction in tumour size compared with approximately 50% of patients who had received chemotherapy [9]. In patients with advanced gastric cancer, tumour size is an independent prognostic factor for the 5-year survival rate [19]. The median best percentage change from baseline in the sum of diameters for all target lesions in this study was − 24.7%, and this reduction may explain the longer OS and rwPFS with T-DXd compared with other therapies used for third- or later-line treatment in patients with advanced gastric cancer. Indeed, the subgroup analysis revealed that a greater reduction in best percent change from baseline in sum of diameters for all target lesions was associated with a longer median OS. These data highlight the significance of tumor shrinkage even in the third or later line treatment in advanced gastric cancer.

In DESTINY-Gastric01, the percentage of patients with an objective response was higher among those with a HER2 expression status of IHC3 + than among those with a HER2 expression status of IHC2 + /ISH [9]. Similar findings were noted in the subgroup analysis for OS, rwPFS and ORR for target lesions in this study. In the Japanese real-world study of T-DXd, patients who had a trastuzumab-free interval of ≥ 8 months (vs. < 8 months) had a longer median OS and PFS, which is in agreement with this study [17]. Patients who had a BOR of PR (vs. SD) in the treatment history of trastuzumab had a significantly longer rwPFS. Thus, the findings of this study could help identify patients who are likely to respond to T-DXd. Nivolumab is a key drug for advanced gastric cancer and can be used as third- or later line therapy for patients with HER2-positive gastric cancer. The treatment sequence with T-DXd and nivolumab is a thus clinical question. In this regard, the current study demonstrated that there was no significant effect of nivolumab treatment at previous therapy on OS, rwPFS, and ORR for target lesions. Caution should be exercised in interpreting the results of this study due to bias in patient selection. In addition, the subgroup analysis revealed that patients with ECOG PS 0–1 (vs. ≥ 2), intestinal type (vs. diffuse type) of primary lesions, without (vs. with) ascites and eligible (vs. ineligible) for participation in DESTINY-Gastric01 had a significantly longer OS and rwPFS. However, these poor prognostic factors are not limited to T-DXd. In a real-world study evaluating the effectiveness of nivolumab in Japan, a multivariate regression analysis revealed that OS and PFS were shorter in patients with ECOG PS 2, histology of diffuse type, peritoneal metastases and ascites compared with patients who had PS 0, non-diffuse type of histology, without peritoneal metastasis and without ascites, respectively [14]. Further studies are thus needed to determine the ideal treatment options for this population.

Although no new grade ≥ 3 AEs of concern were detected in this real-world study, a higher percentage of patients discontinued T-DXd due to AEs in this study (23.7%) compared with the clinical trials (14.0–15.2%) [8, 9]. The approved dosage of T-DXd for patients with gastric cancer is 6.4 mg/kg every 3 weeks; however, 78.2% of patients in this study received T-DXd at > 5.4– ≤ 6.4 mg/kg as initial dose in the real-world setting. From cycle 2 onwards, the proportion of patients receiving T-DXd at a dose of > 5.4– ≤ 6.4 mg/kg reduced, whereas the proportion of those receiving > 4.4– ≤ 5.4 mg/kg increased. The reasons for the lower dosage of T-DXd in this study compared with DESTINY-Gastric01 could be patients’ poor health condition as indicated by the ECOG PS and rather prudent attitude of physicians who prescribed T-DXd outside the clinical trial setting [9]. In the current study, OS and rwPFS increased significantly among patients who continued T-DXd treatment beyond the 3rd cycle and experienced grade ≥ 3 AEs or experienced AEs leading to dose reduction or interruption, or discontinuation compared with those who did not experience these AEs or withdrew treatment before the 3rd cycle. This may suggest that patients who are able to continue treatment and tolerate AEs are likely to show better treatment outcome with T-DXd probably due to a higher concentration of T-DXd in the systemic and local circulation, which warrants further investigation. Nonetheless, this observation implies that it might be clinically important to avoid the dose reduction without significant toxicity. In patients with ECOG PS 0/1 and without poor prognostic factors, it thus seems better to initiate T-DXd dosing at 6.4 mg/kg whenever possible and the dose should be reduced if patients experience AEs. On the other hand, the optimal T-DXd dosing for a population whose treatment was discontinued within fewer than 3 cycles, including those with poor PS, cannot be determined, which warrants further study.

The current study has several limitations. First, this was an observational study with no comparator group. A comparator group could further help in contextualising the findings of this study. Second, lesion assessment and timing of imaging were not defined in this study, and central review was not performed for tumour response evaluation. Third, the median follow-up time was limited (less than actual OS). Fourth, as this study was conducted in Japan, the findings have limited generalisability and must be interpreted with caution when generalising to other populations/countries.

In conclusion, patients treated with T-DXd had a longer OS and higher ORR than those in previously reported clinical studies of third- or later-line treatment regimens for gastric cancer and confirms the effectiveness of T-DXd in real-world clinical practice. The safety of T-DXd was comparable with that reported in clinical trials, and no new safety signals were identified. Thus, T-DXd is effective and has a manageable safety profile as a third- or later-line treatment for patients with HER2-positive gastric or GEJ cancer in clinical practice.

## Supplementary Information

Below is the link to the electronic supplementary material.

Supplementary file1 (DOCX 3246 KB)
