# Correction: Real-world effectiveness and safety of trastuzumab-deruxtecan in Japanese patients with HER2-positive advanced gastric cancer (EN-DEAVOR study)

**DOI:** 10.1007/s10120-024-01570-x

**Published:** 2024-11-28

**Authors:** Hisato Kawakami, Koki Nakanishi, Akitaka Makiyama, Hirotaka Konishi, Satoshi Morita, Yukiya Narita, Naotoshi Sugimoto, Keiko Minashi, Motohiro Imano, Rin Inamoto, Yasuhiro Kodera, Hiroki Kume, Keita Yamaguchi, Wataru Hashimoto, Kei Muro, EN-DEAVOR Study Group

**Affiliations:** 1 Department of Medical Oncology, Kindai University Faculty of Medicine, Osakasayama, Japan; 2 Department of Gastroenterological Surgery, Nagoya University Graduate School of Medicine, Nagoya, Japan; 3 Cancer Center, Gifu University Hospital, Gifu, Japan; 4 Division of Digestive Surgery, Kyoto Prefectural University of Medicine, Kyoto, Japan; 5 Department of Biomedical Statistics and Bioinformatics, Kyoto University Graduate School of Medicine, Kyoto, Japan; 6 Department of Clinical Oncology, Aichi Cancer Center Hospital, Nagoya, Japan; 7 Department of Genetic Oncology, Osaka International Cancer Institute, Osaka, Japan; 8 Division of Gastroenterology, Chiba Cancer Center, Chiba, Japan; 9 Department of Surgery, Kindai University Faculty of Medicine, Osakasayama, Japan; 10 Department of Gastroenterology, Saitama Cancer Center, Saitama, Japan; 11 Oncology Medical Science Department I, Daiichi Sankyo Co. Ltd., Tokyo, Japan; 12 Data Intelligence Department, Daiichi Sankyo Co. Ltd., Tokyo, Japan

**Correction: Gastric Cancer** 10.1007/s10120-024-01555-w

The funding information section was missing from this article and should have read “This study was funded by Daiichi Sankyo Co. Ltd., Japan.”.

The original article has been corrected.

