# Supplementary material for: Real-world effectiveness and safety of trastuzumab-deruxtecan in Japanese patients with HER2-positive advanced gastric cancer (EN-DEAVOR study)
Source: Gastric Cancer. 2024 Oct 10;28(1):51–61. doi: 10.1007/s10120-024-01555-w (PMC11706843; doi:10.1007/s10120-024-01555-w)
Supplement: Supplementary file 1 — Supplementary file1 (DOCX 3246 KB) [file 10120_2024_1555_MOESM1_ESM.docx]

**Supplementary Material**

**Supplementary Table 1.** Inclusion criteria for DESTINY-Gastric01

| **Characteristic** | **Description** |
| --- | --- |
| ECOG PS | 0, 1 |
| Platelets (x 10^4^/µL) | ≥10 |
| Haemoglobin (g/dL) | ≥8 |
| Neutrophil (/µL) | ≥1500 |
| Creatinine clearance (mL/min) | ≥30 |
| AST/ALT | Male: ≤9 (liver metastasis: ≤15)  Female: ≤12.9 (liver metastasis: ≤21.5) |
| Total bilirubin | ≤2.25 (liver metastasis: <4.5) |
| Albumin (g/dL) | ≥2.5 |
| Metastasis site: Brain | None |
| Ascites (high) | None |
| Target lesion | Yes |
| Previous therapy: Trastuzumab treatment | Yes |

ALT, alanine transaminase; AST, aspartate aminotransferase; ECOG PS, Eastern Cooperative Oncology Group performance status

Ref: Shitara K, Bang YJ, Iwasa S, Sugimoto N, Ryu MH, Sakai D, et al. Trastuzumab deruxtecan in previously treated HER2-positive gastric cancer. N Engl J Med. 2020;382:2419–‍30. https://doi.org/10.1056/NEJMoa2004413

**Supplementary Table 2.** Objective response rate and best overall response in patients with a target lesion (all patients)

|  | **n (%)** | **95% CI^a^** |
| --- | --- | --- |
| **Presence or absence of target lesion: Yes** |  |  |
| Response | 97 (42.9) | 36.4–49.7 |
| Non-response | 129 (57.1) |  |
| **Presence or absence of target lesions: Yes** |  |  |
| CR | 5 (2.2) | 0.7–5.1 |
| PR | 92 (40.7) | 34.2–47.4 |
| SD | 87 (38.5) | 32.1–45.2 |
| PD | 31 (13.7) | 9.5–18.9 |
| NE^b^ | 11 (4.9) | 2.5–8.5 |

^a^Determined using the Clopper-Pearson method

^b^Including non-implemented

CI, confidence interval; CR, complete response; NE, not evaluable; PD, progressive disease; PR, partial response; SD, stable disease

**Supplementary Table 3** AEs leading to dose reduction, interruption or discontinuation of T-DXd

|  | **Dose reduction, interruption or discontinuation** | **Dose reduction** | **Dose interruption** | **Discontinuation** |
| --- | --- | --- | --- | --- |
| Any AE | 190 (60.9) | 115 (36.9) | 106 (34.0) | 74 (23.7) |
| Haematotoxicity | 97 (31.1) | 61 (19.6) | 72 (23.1) | 4 (1.3) |
| Non-haematotoxicity | 139 (44.6) | 67 (21.5) | 48 (15.4) | 74 (23.7) |
| Nausea | 29 (9.3) | 19 (6.1) | 8 (2.6) | 10 (3.2) |
| Vomiting | 9 (2.9) | 5 (1.6) | 3 (1.0) | 4 (1.3) |
| Anorexia | 55 (17.6) | 39 (12.5) | 16 (5.1) | 19 (6.1) |
| Malaise | 47 (15.1) | 32 (10.3) | 15 (4.8) | 11 (3.5) |
| Anaemia | 17 (5.4) | 9 (2.9) | 9 (2.9) | 3 (1.0) |
| Diarrhoea | 11 (3.5) | 9 (2.9) | 5 (1.6) | 1 (0.3) |
| Constipation | 3 (1.0) | 0 (0.0) | 3 (1.0) | 0 (0.0) |
| Fatigue | 12 (3.8) | 9 (2.9) | 3 (1.0) | 3 (1.0) |
| Neutrophil count decreased | 76 (24.4) | 49 (15.7) | 60 (19.2) | 1 (0.3) |
| Platelet count decreased | 15 (4.8) | 10 (3.2) | 8 (2.6) | 1 (0.3) |
| White blood cell decreased | 13 (4.2) | 8 (2.6) | 8 (2.6) | 0 (0.0) |
| Febrile neutropenia | 2 (0.6) | 0 (0.0) | 1 (0.3) | 1 (0.3) |
| Interstitial pneumonia | 29 (9.3) | 0 (0.0) | 0 (0.0) | 29 (9.3) |
| Pneumonitis | 7 (2.2) | 0 (0.0) | 1 (0.3) | 6 (1.9) |
| Others | 34 (10.9) | 5 (1.6) | 21 (6.7) | 17 (5.4) |

Data are presented as n (%)

AE, adverse event; T-DXd, trastuzumab-deruxtecan

**Subgroup analysis**

**Supplementary Fig. 1** Subgroup analysis of rwPFS


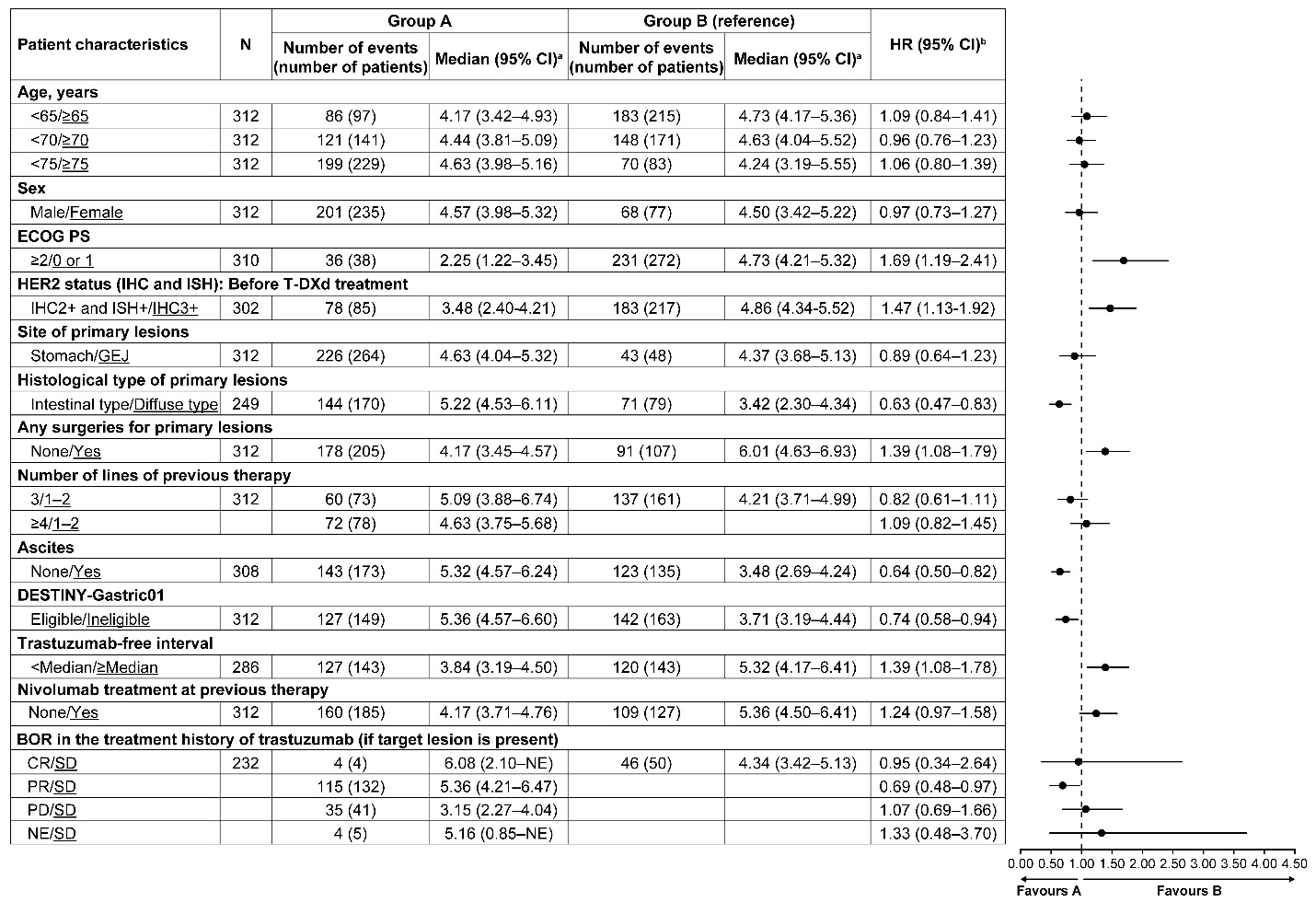


The underlined values are for Group B (reference)

^a^Brookmeyer and Crowley method

^b^Using the univariate Cox’s proportional hazard model

BOR, best overall response; CI, confidence interval; CR, complete response; ECOG PS, Eastern Cooperative Oncology Group performance status; GEJ, gastroesophageal junction; HER2, human epidermal growth factor receptor type 2; HR, hazard ratio; IHC, immunohistochemistry; ISH, in situ hybridisation; NE, not estimable; PD, progressive disease; PR, partial response; rwPFS, real-world progression-free survival; SD, stable disease; T-DXd, trastuzumab deruxtecan

**Supplementary Fig. 2** Subgroup analysis of ORR in patients with target lesions


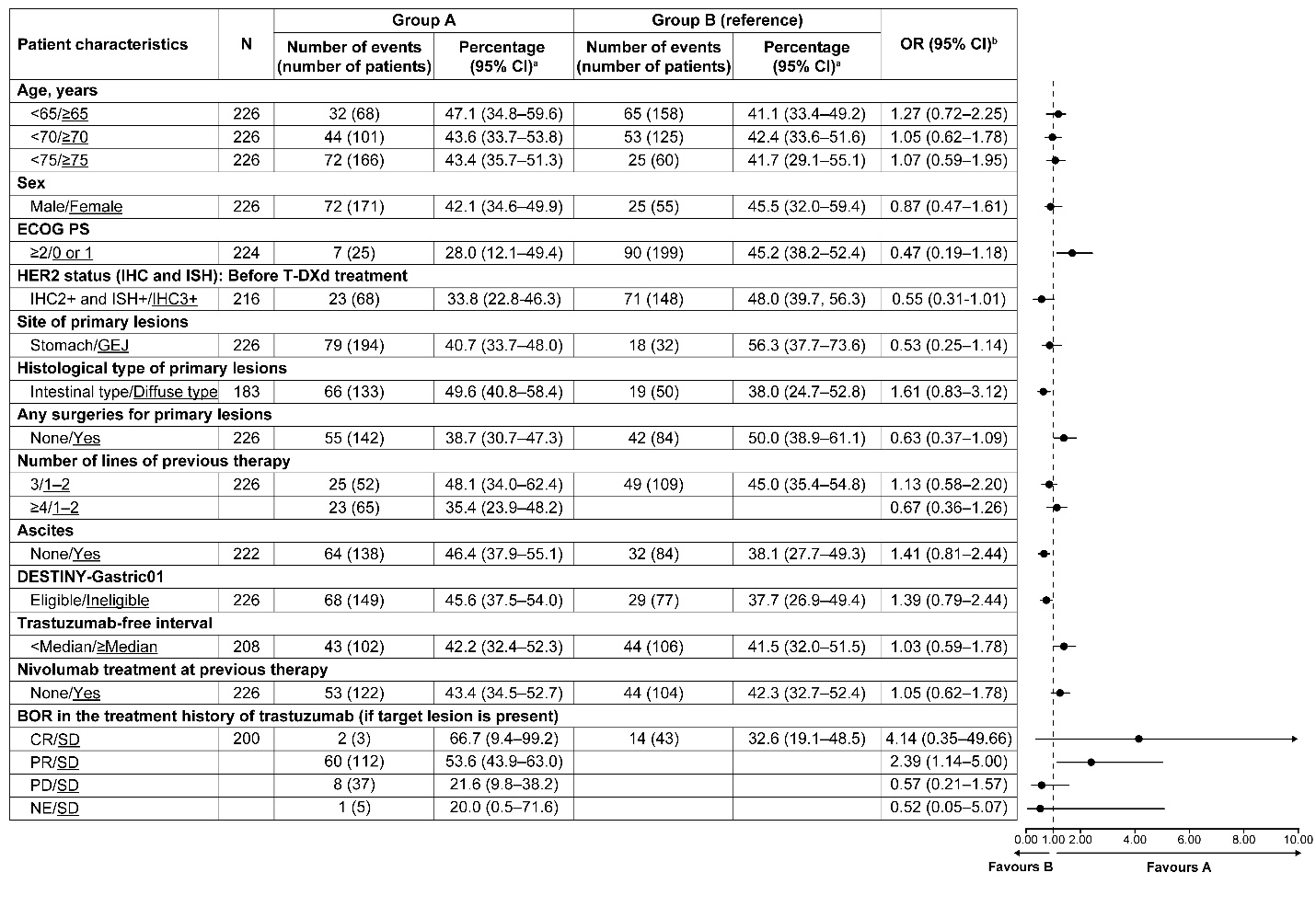


The underlined values are for Group B (reference)

^a^Brookmeyer and Crowley method

^b^Using the univariate Cox’s proportional hazard model

BOR, best overall response; CI, confidence interval; CR, complete response; ECOG PS, Eastern Cooperative Oncology Group performance status; GEJ, gastroesophageal junction; HER2, human epidermal growth factor receptor type 2; IHC, immunohistochemistry; ISH, in situ hybridisation; NE, not estimable; OR, odds ratio; ORR, objective response rate; PD, progressive disease; PR, partial response; SD, stable disease; T-DXd, trastuzumab deruxtecan

**Supplementary Fig. 3** Subgroup analysis of OS by best percentage change in the sum of diameters of all target lesions

**
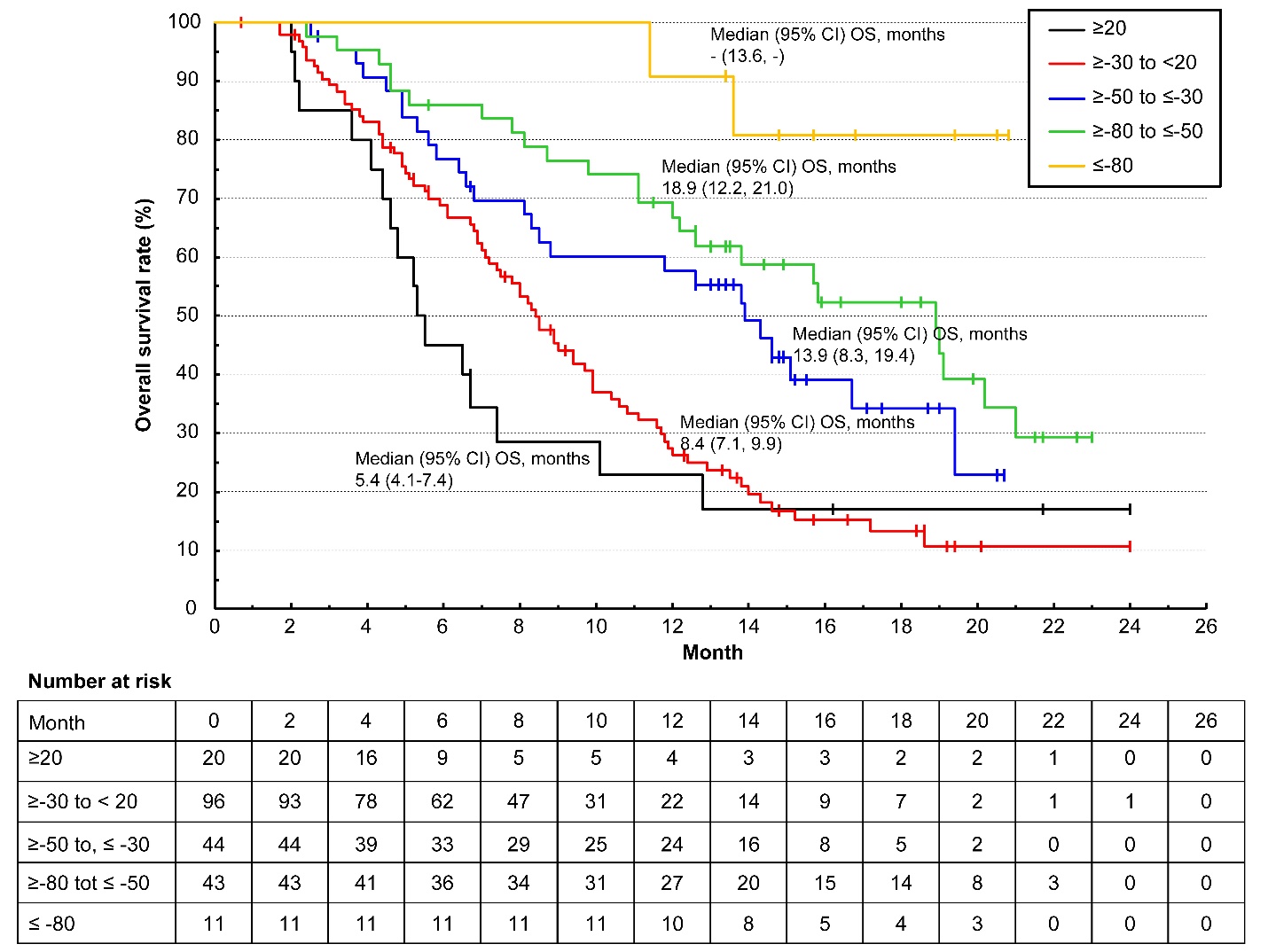
**

The Brookmeyer and Crowley method was used to calculate the median and 95% CICI, confidence interval; OS, overall survival

**Effectiveness and safety of T-DXd in the PI-compliant population**

*Effectiveness outcomes*

A total of 214 patients comprised the PI-compliant population. The median (95% CI) OS, rwPFS and TTF (months) was 9.1 (8.0–11.7), 4.2 (3.8–4.9) and 3.8 (3.3–4.2), respectively (Supplementary Fig. 4). Overall, 33.2% of patients had an objective response; the response rate was 44.8% and 3.3% in patients with and without a target lesion, respectively.

**Supplementary Fig. 4** Survival outcomes of T-DXd in the PI-compliant population: **(a)** OS, **(b)** rwPFS and **(c)** TTF


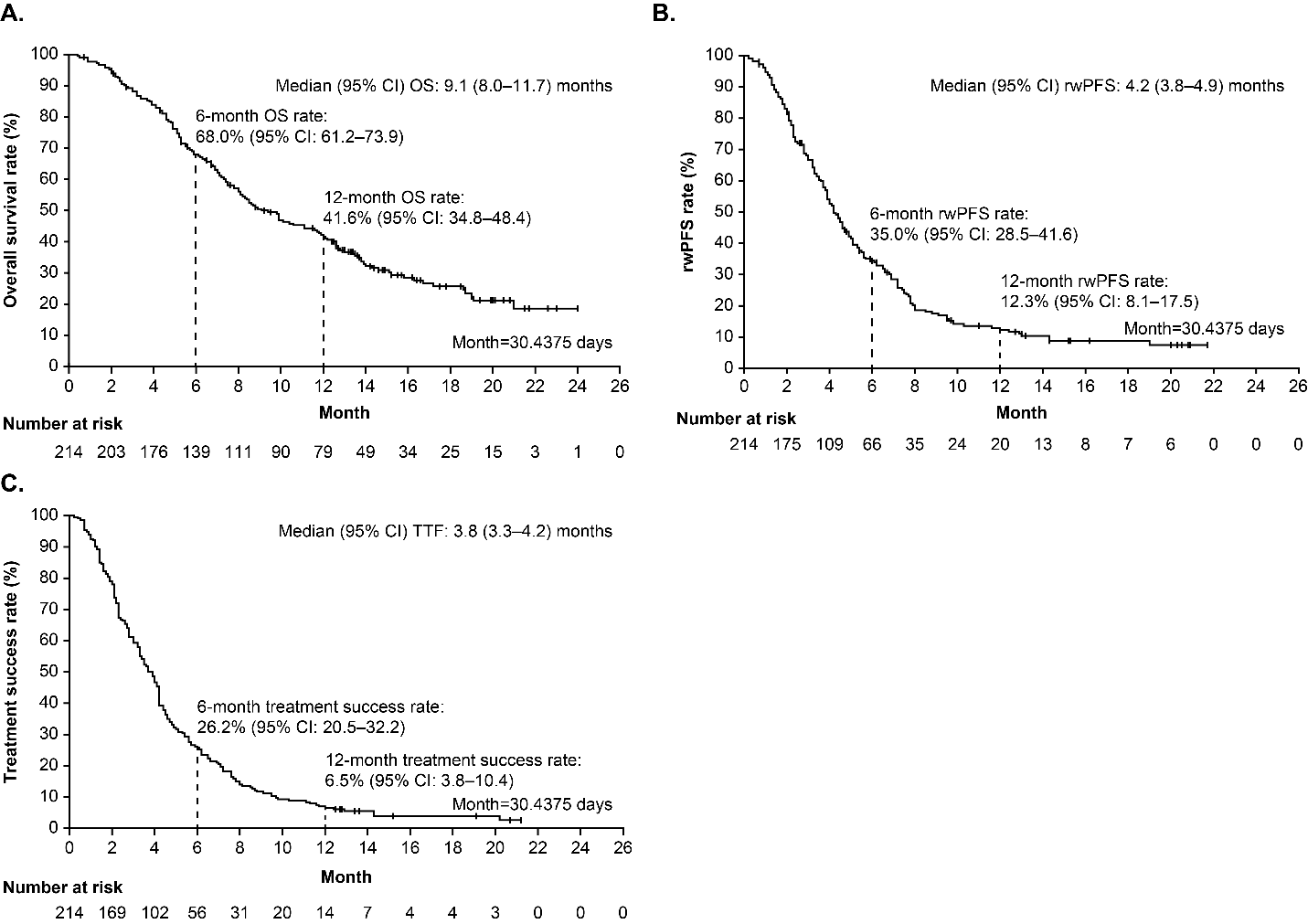


The median (95% CI) time was determined using the Brookmeyer and Crowley method

CI, confidence interval; OS, overall survival; rwPFS, real-world progression-free survival; TTF, time to treatment failure; T-DXd, trastuzumab-deruxtecan

The response rate was 44.8% in patients with a target lesion (n=154); the best overall response was complete response (CR) in 1.3%, partial response (PR) in 43.5%, stable disease (SD) in 34.4%, progressive disease (PD) in 16.2% and not estimable in 4.5% of patients (Supplementary Table 4).

**Supplementary Table 4.** Objective response rate and best overall response in patients with a target lesion (PI-compliant population)

|  | **n (%)** | **95% CI^a^** |
| --- | --- | --- |
| **Presence or absence of target lesion: Yes** |  |  |
| Response | 69 (44.8) | 36.8–53.0 |
| Non-response | 85 (55.2) |  |
| **Presence or absence of target lesions: Yes** |  |  |
| CR | 2 (1.3) | 0.2–4.6 |
| PR | 67 (43.5) | 35.5–51.7 |
| SD | 53 (34.4) | 27.0–42.5 |
| PD | 25 (16.2) | 10.8–23.0 |
| NE^b^ | 7 (4.5) | 1.8–9.1 |

^a^Determined using the Clopper-Pearson method.

^b^Including non-implemented

CI, confidence interval; CR, complete response; NE, not evaluable; PD, progressive disease; PI, package insert; PR, partial response; SD, stable disease

The median (range) sum of diameters of the baseline tumour was 49.6 (11.0–229.7) mm, and the best percentage change from baseline in the sum of diameters for all target lesions was −26.7% (−100.0 to 127.7; Supplementary Fig. 5).

**Supplementary Fig. 5** Best percentage change from baseline in the sum of diameters of the target lesion (PI-compliant population)


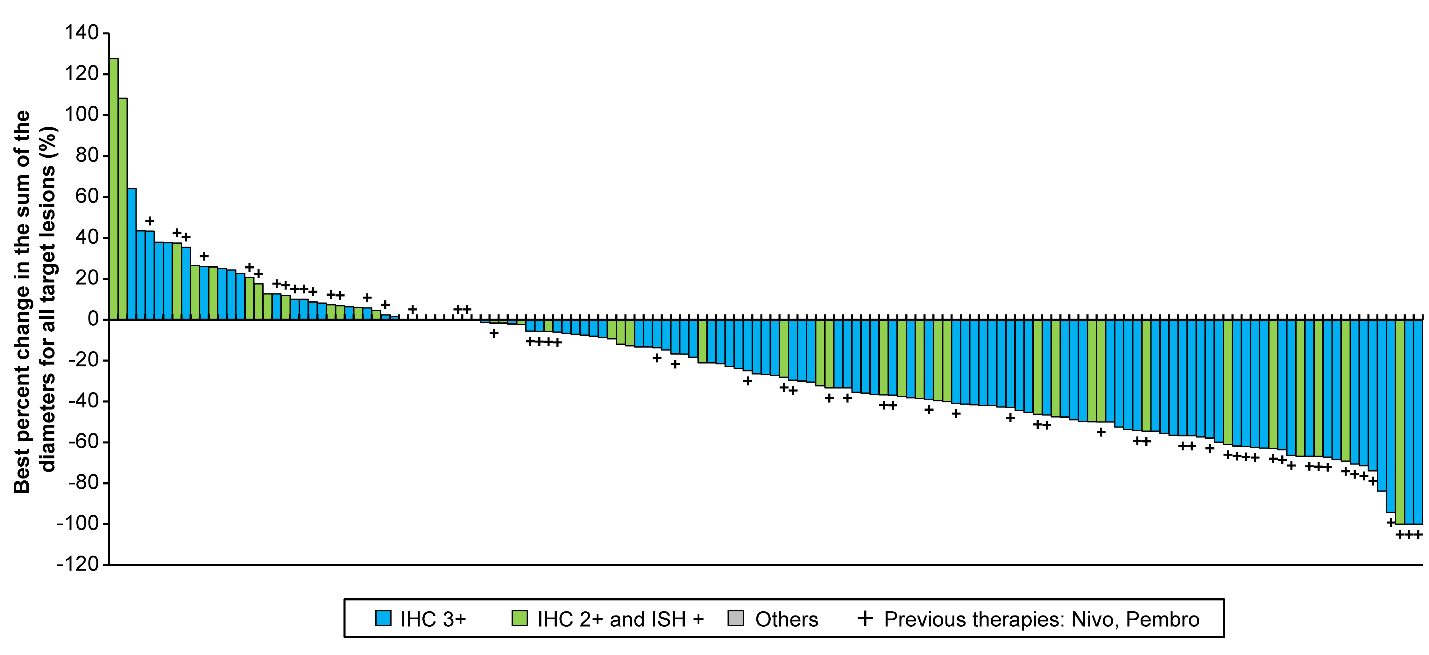


IHC, immunohistochemistry; ISH, in situ hybridisation; Nivo, nivolumab; Pembro, pembrolizumab; PI, package insert

*Subgroup analysis*

Patients with ECOG PS ≥2, HER2 IHC 2+ and ISH+ and trastuzumab-free interval <median had a significantly shorter OS and rwPFS compared with those who had ECOG PS 0 or 1, HER2 IHC 3+ and trastuzumab-free interval ≥median, respectively. In addition, patients aged <65 years had a significantly shorter OS compared with those aged ≥65 years. Patients with intestinal type of primary lesions, without ascites, and those eligible for participation in DESTINY-Gastric01 had a significantly longer OS and rwPFS than those with diffuse lesions and ineligible for participation in DESTINY-Gastric01, respectively (Supplementary Figs. 6 and 7). Patients without ascites had significantly better ORR for target lesions than those without ascites (Supplementary Fig. 8).

**Supplementary Fig. 6** Subgroup analysis of OS in PI-compliant population


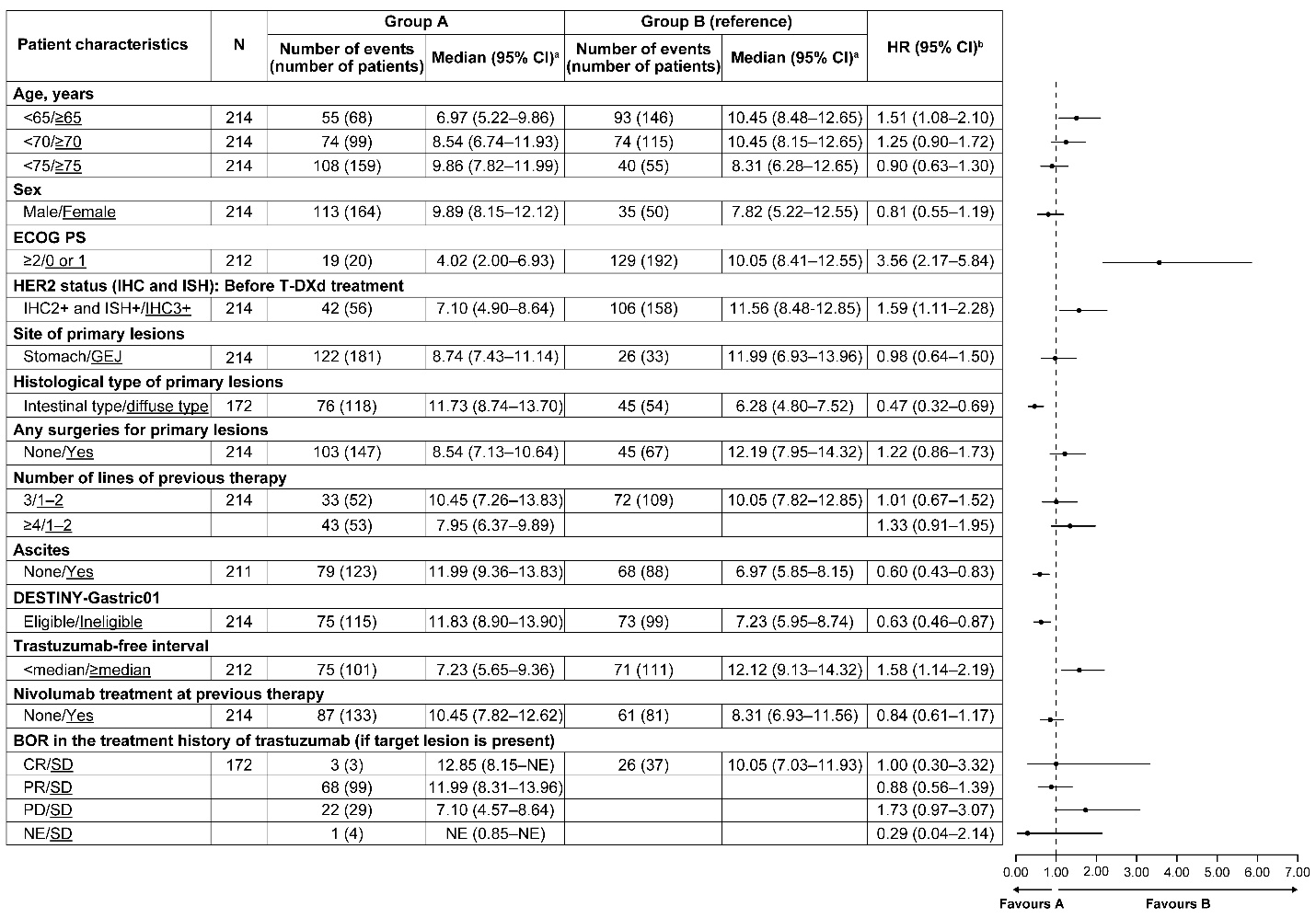


The underlined values are for Group B (reference)

^a^Brookmeyer and Crowley method

^b^Using the univariate Cox’s proportional hazard model

BOR, best overall response; CI, confidence interval; CR, complete response; ECOG PS, Eastern Cooperative Oncology Group performance status; GEJ, gastroesophageal junction; HER2, human epidermal growth factor receptor type 2; HR, hazard ratio; IHC, immunohistochemistry; ISH, in situ hybridisation; NE, not estimable; OS, overall survival; PD, progressive disease; PI, package insert; PR, partial response; SD, stable disease; T-DXd, trastuzumab deruxtecan

**Supplementary Fig.7** Subgroup analysis of rwPFS in PI-compliant population


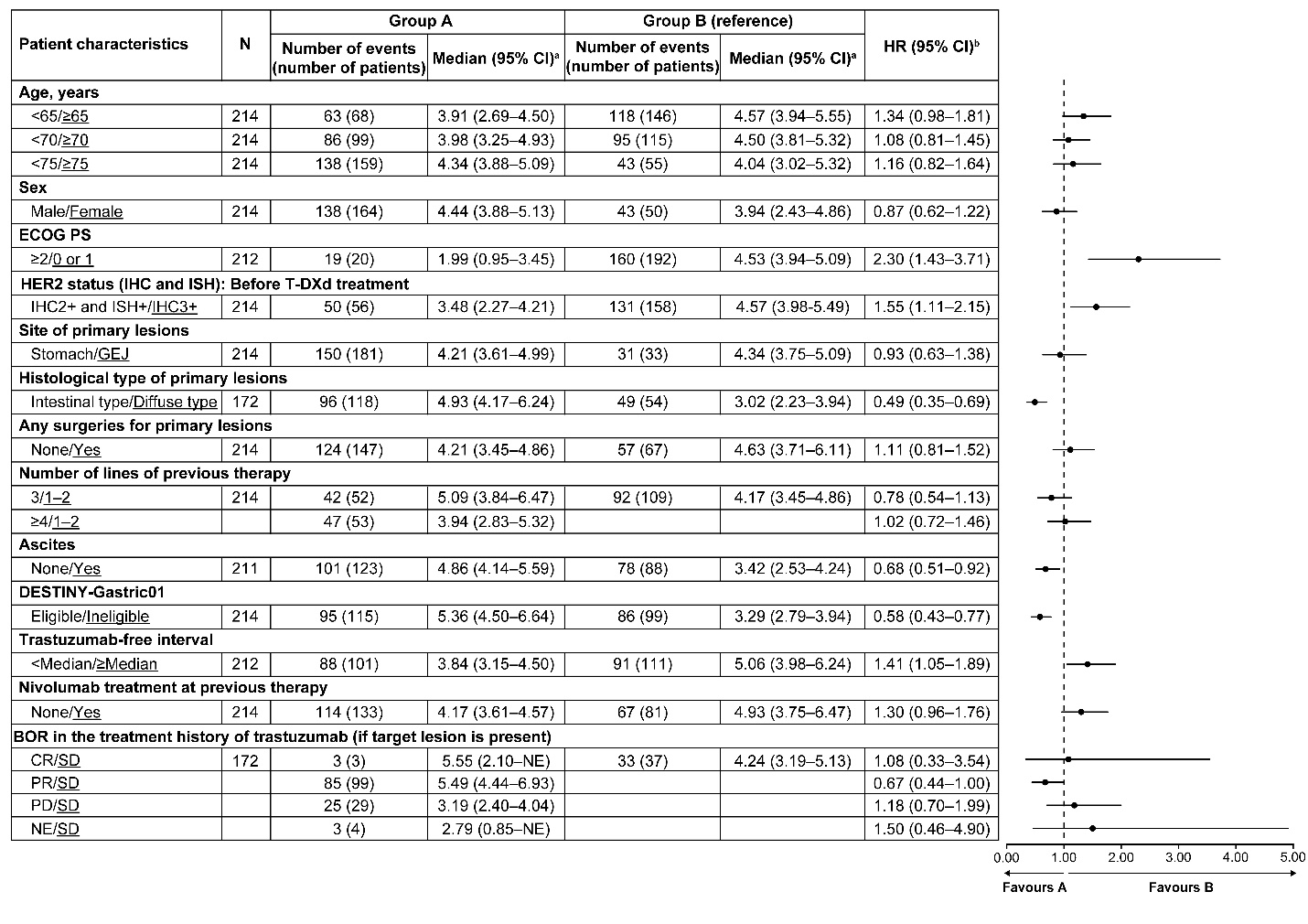


The underlined values are for Group B (reference)

^a^Brookmeyer and Crowley method

^b^Using the univariate Cox’s proportional hazard model

BOR, best overall response; CI, confidence interval; CR, complete response; ECOG PS, Eastern Cooperative Oncology Group performance status; GEJ, gastroesophageal junction; HER2, human epidermal growth factor receptor type 2; HR, hazard ratio; IHC, immunohistochemistry; ISH, in situ hybridisation; NE, not estimable; PD, progressive disease; PI, package insert; PR, partial response; rwPFS, real-world progression‑free survival; SD, stable disease; T-DXd, trastuzumab deruxtecan

**Supplementary Fig. 8** Subgroup analysis of ORR in patients with target lesions in PI‑compliant population


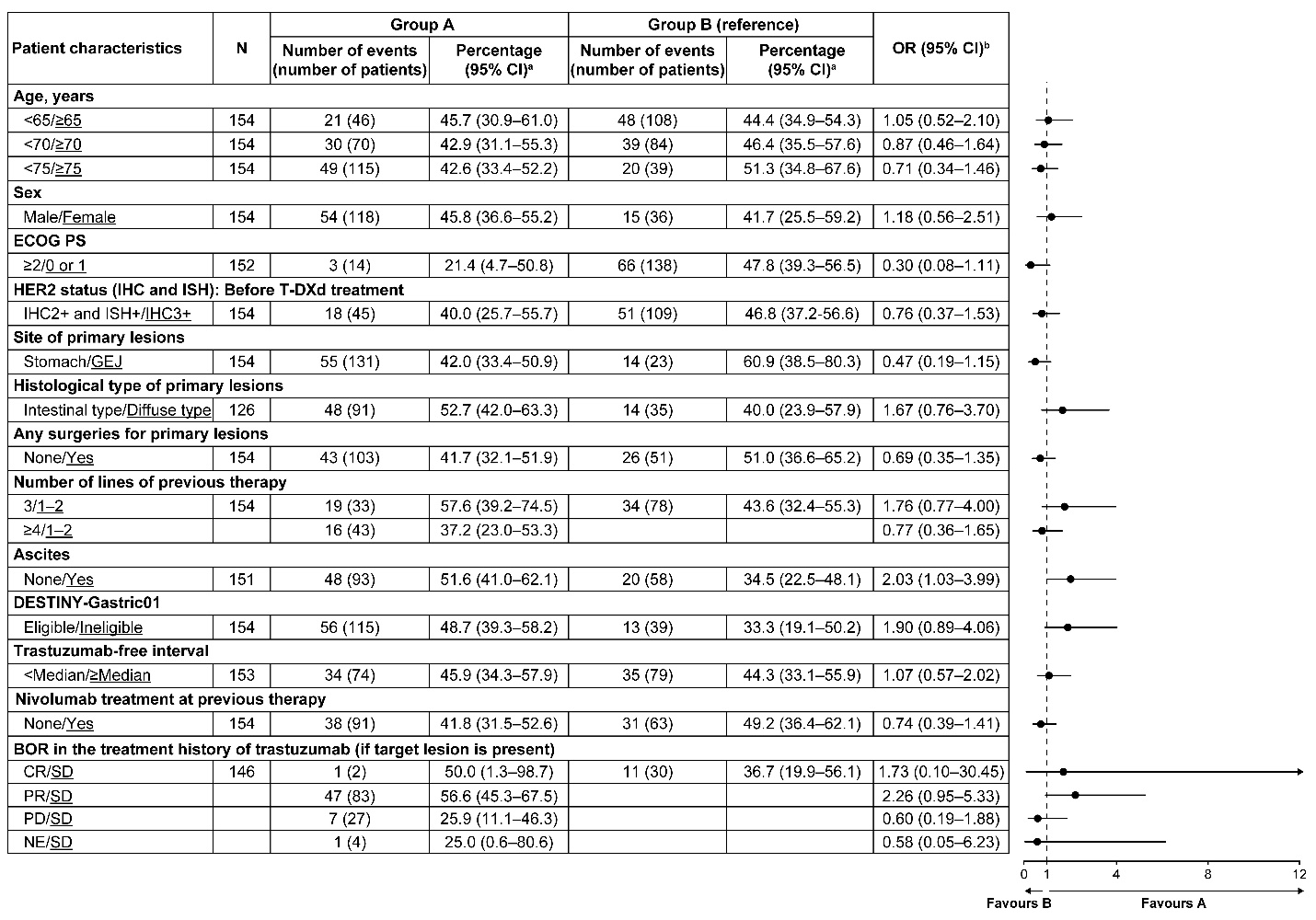


The underlined values are for Group B (reference)

^a^Brookmeyer and Crowley method

^b^Using the univariate Cox’s proportional hazard model

BOR, best overall response; CI, confidence interval; CR, complete response; ECOG PS, Eastern Cooperative Oncology Group performance status; GEJ, gastroesophageal junction; HER2, human epidermal growth factor receptor type 2; IHC, immunohistochemistry; ISH, in situ hybridisation; NE, not estimable; OR, odds ratio; ORR, objective response rate; PD, progressive disease; PI, package insert; PR, partial response; SD, stable disease; T-DXd, trastuzumab deruxtecan

At the 2-year follow-up, the median OS and rwPFS were significantly longer among those who experienced AEs that led to dose reduction, interruption or discontinuation after having completed 3 cycles of T-DXd compared with those who did not experience these AEs (Supplementary Figs. 9A and 9B). The median rwPFS but not OS was significantly longer for patients who experienced grade ≥3 AEs after having completed 3 cycles of T-DXd compared with those who did not experience these AEs (Supplementary Figs. 9C and 9D).

**Supplementary Fig. 9** Survival rates of patients requiring dose adjustment and those who experienced grade ≥3 AEs after having completed 3 cycles of T-DXd (PI-compliant population)
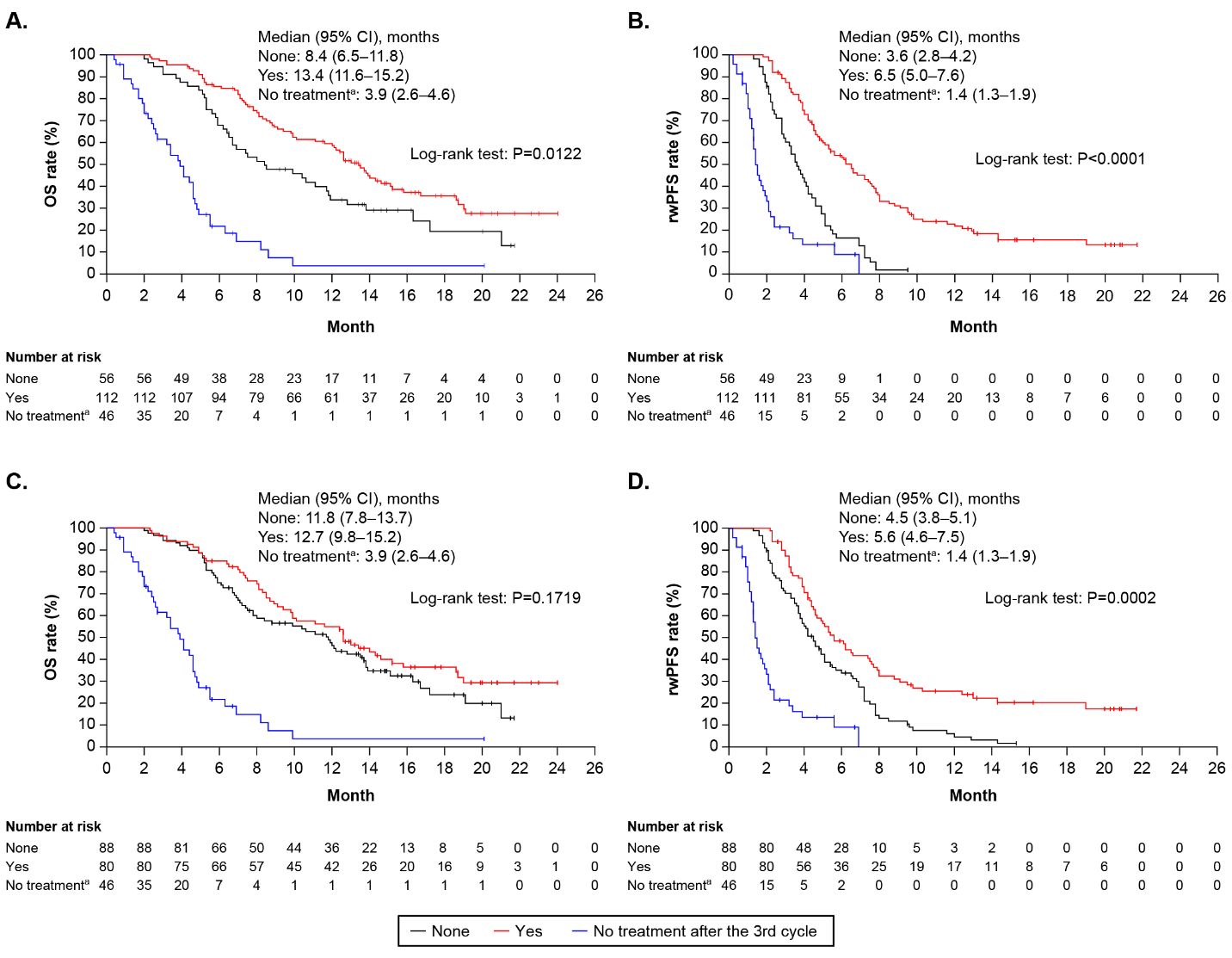


A. OS in patients who experienced AEs that led to dose reduction, postponement, or discontinuation of T-DXd under T-DXd treatment, B. rwPFS in patients who experienced AEs that led to dose reduction, postponement, or discontinuation of T-DXd under T-DXd treatment, C. OS in patients who experienced grade ≥3 AEs under T-DXd treatment, D. rwPFS in patients who experienced grade ≥3 AEs under T-DXd treatment.

1 month=30.4375 days

^a^T-DXd was discontinued within 2 cycles

AE, adverse event; CI, confidence interval; OS, overall survival; PI, package insert; rwPFS, real‑world progression-free survival; T‑DXd, trastuzumab-deruxtecan
